# HEM1 Actin Immunodysregulatory Disorder: Genotypes, Phenotypes, and Future Directions

**DOI:** 10.1007/s10875-022-01327-0

**Published:** 2022-07-22

**Authors:** Sarah Cook, Michael J. Lenardo, Alexandra F. Freeman

**Affiliations:** 1 Molecular Development of the Immune System Section, Laboratory of Immune System Biology, and Clinical Genomics Program, Division of Intramural Research, National Institute of Allergy and Infectious Diseases, National Institutes of Health, Bethesda, MD USA; 2 Present Address: Vanderbilt University School of Medicine, Nashville, TN USA; 3 Laboratory of Clinical Immunology and Microbiology, Division of Intramural Research, National Institute of Allergy and Infectious Diseases, National Institutes of Health, Bethesda, MD USA

**Keywords:** Actinopathy, NCKAP1L, WRC, Autoimmunity, Autoinflammation, HLH, SLE

## Abstract

Cells of the innate and adaptive immune systems depend on proper actin dynamics to control cell behavior for effective immune responses. Dysregulated actin networks are known to play a pathogenic role in an increasing number of inborn errors of immunity. The WAVE regulatory complex (WRC) mediates branched actin polymerization, a process required for key cellular functions including migration, phagocytosis, vesicular transport, and immune synapse formation. Recent reports of pathogenic variants in *NCKAP1L*, a hematopoietically restricted gene encoding the HEM1 protein component of the WRC, defined a novel disease involving recurrent bacterial and viral infections, autoimmunity, and excessive inflammation (OMIM 141180). This review summarizes the diverse clinical presentations and immunological phenotypes observed in HEM1-deficient patients. In addition, we integrate the pathophysiological mechanisms described in current literature and highlight the outstanding questions for diagnosis and management of the HEM1 actin immunodysregulatory disorder.

## Introduction

Dynamic control of actin polymerization is crucial for cellular migration, adhesion, division, membrane transport, and spatiotemporal coordination of signals that govern metabolism and proliferation [1, 2]. Cells of the innate and adaptive immune systems depend on proper actin dynamics to control effective responses, which is highlighted by inborn errors of immunity (IEI) affecting actin networks, known as actinopathies (reviewed in [3–7]). Dysregulated actin networks cause recurrent infections often coupled with autoinflammation, autoimmunity, and atopic disease in IEIs such as Wiskott Aldrich syndrome, DOCK8 deficiency, and X-linked moesin-associated immune deficiency [3]. Recently, three reports investigating novel mutations in *NCKAP1L*, a hematopoietically restricted gene encoding the HEM1 protein component of the WAVE regulatory complex (WRC), established how improper actin networks lead to a clinical syndrome involving recurrent bacterial and viral infections and autoimmunity (OMIM 141180) [8–10]. One study showed how loss of the WRC, one of the two primary actin-related proteins 2/3 (Arp2/3) nucleation promoting factors (NPF), contributes to the complex picture of impaired specific immunity and concomitant nonspecific immune system hyperactivation [8]. In this review, we summarize the clinical manifestations and disease pathogenesis of HEM1 deficiency and propose future directions to improve therapy.

## HEM1 and Branched Actin Polymerization

**Fig. 1:**
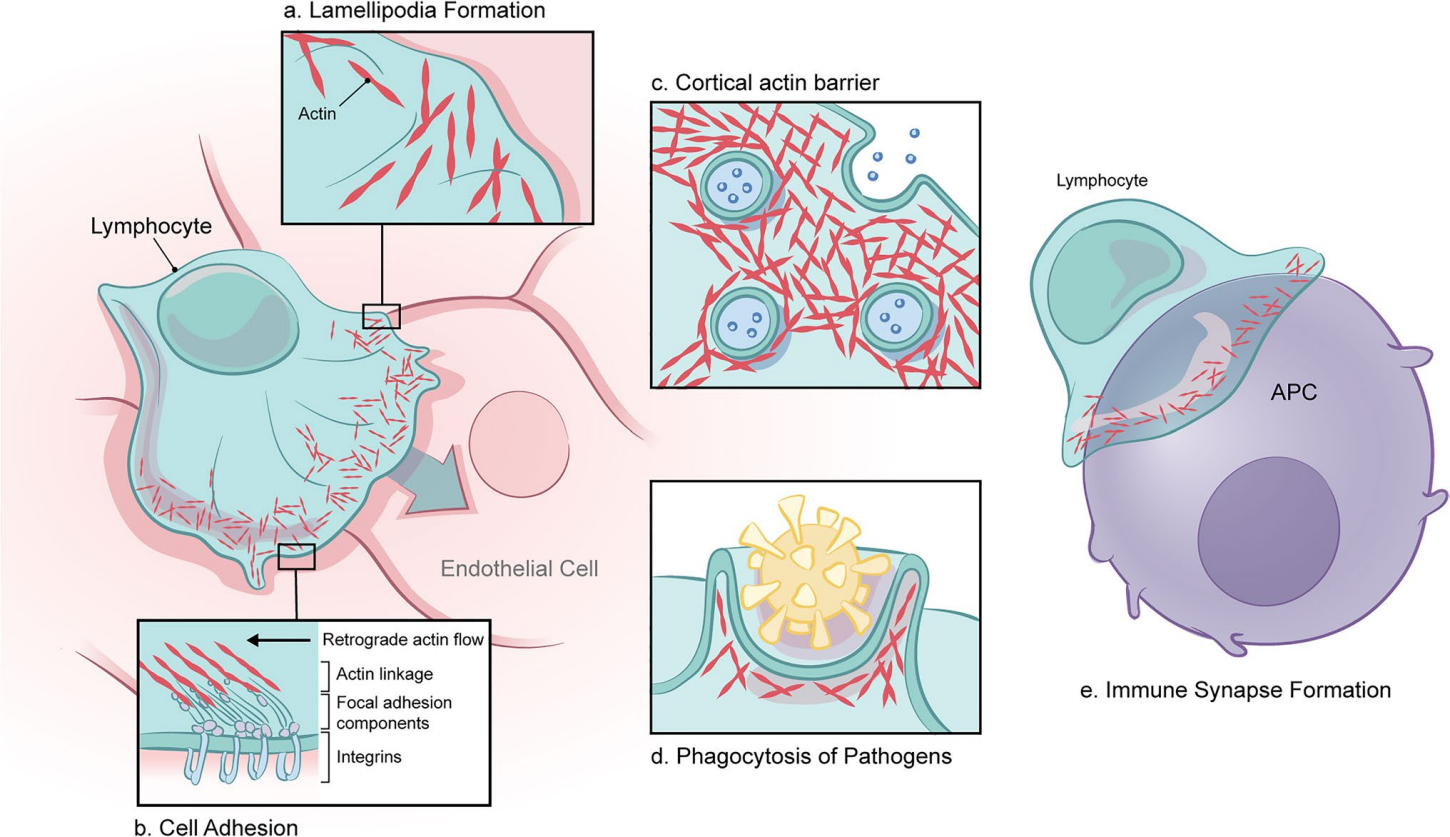
Critical functions of branched actin in immune cells. (**a**) Sheets of branched actin protrude to form lamellipodia at the leading edge of migrating cells, guiding transport through extracellular matrices (ECM). (**b**) While the extracellular domains of integrin proteins bind to ligands in the ECM during migration or on neighboring cells during adhesion, their intracellular domains recruit components of focal adhesion complexes that bind to the cytoskeleton. Retrograde actin flow, where newly assembled actin pushes against the membrane and filaments elongate toward the cell center, generates tensile strength for integrin activation, focal adhesion maturation, and cell migration. (**c**) A ring of cortical branched actin below the cell membrane mechanically inhibits vesicle fusion and allows for coordinated exocytosis. (**d**) Branched actin is required for extracellular pathogen phagocytosis by innate immune cells. (**e**) At immune synapses between lymphocytes and antigen presenting cells, a ring of peripheral branched actin drives cell spreading across the activating surface

Actin networks are critical for kinetic processes in nearly all eukaryotic cells. Globular actin (G-actin) monomers are incorporated into either linear or branching polymers. In human immune cells, formin proteins polymerize G-actin into linear filamentous actin (F-actin) bundles to generate filopodia, which are protruding structures required for chemosensation. By contrast, branched actin networks are required for cell spreading and immune synapse formation, lamellipodia formation, endo- and exocytosis, and phagocytosis (Fig. 1). Additionally, a dense ring of branched actin surrounds the cytosol, creating a barrier at the cell cortex that regulates vesicle secretion [11–13]. Branched actin is polymerized by the ARP2/3 complex after stimulation by the Wiskott-Aldrich Syndrome protein (WASp) family of actin NPFs, which includes the WASp family verprolin homologous (WAVE) proteins. WAVE isoforms are the scaffold of the WRC, an obligate heteropentameric complex containing the additional subunits: hematopoietic protein 1/2 (HEM1/2), cytoplasmic FMR1 interacting protein 1/2 (CYFIP1/2), hematopoietic stem/progenitor cell protein 300 (HSPC300), and Abelson interactor 1/2 (ABI1/2). Differential RNA expression governs which isoform will be incorporated in the WRC [14]. WAVE2 is ubiquitously expressed and is the predominate form in hematopoietic cell lineages, while WAVE1 and WAVE3 expression is limited to neural tissues [15]. HEM1 expression is restricted to hematopoietic cells and its counterpart, HEM2, is expressed in all other tissues [16]. Tissue specificity of subunits suggests that composition of the WRC alters its regulation and function between different cell types.

The HEM1/2 and CYFIP1/2 subunits of the WRC are structurally homologous and form a heterodimer with a flat, polybasic surface that facilitates plasma membrane association [1]. At the membrane, the WRC responds to a panoply of signals from phosphatidylinositol (3,4,5)-triphosphate, Abelson (ABL) and cyclin dependent kinase 5 (CDK5) kinases, and SH3-domain containing scaffolding proteins such as IRSp53 [1]. Importantly, small GTPases that cluster at the plasma membrane can also activate the WRC. Two distinct RAC1-binding sites on CYFIP1/2 and a recently discovered ARF1-binding site on HEM1/2 cooperatively activate the WRC [16, 17]. Combined, these WRC-activating signals initiate ARP2/3-mediated branched actin polymerization to generate lamellipodia at the cell’s leading edge (Fig. 1a) [2]. Tensile force generated by polarized F-actin polymerization allows for integrin activation and focal adhesion maturation, both of which are necessary for cellular adhesion and migration through extracellular matrices (Fig. 1b) [18, 19]. Additionally, branched actin is critical for morphological changes during phagocytosis (Fig. 1d) [20].

Beyond its structural necessity in immune cells, the actin cytoskeleton plays a central role in transmembrane signaling. A dense ring of cortical branched actin below the cell membrane is a barrier that mechanically inhibits nonspecific vesicle fusion, thereby allowing for coordinated exocytosis (Fig. 1c) [11, 13, 21]. ARP2/3-mediated actin polymerization drives cell spreading at immunological synapses and shapes the complex architectures that enable efficient antigen presentation and lymphocyte activation (Fig. 1e) [12, 22, 23]. Upon cellular stimulation, the WRC is recruited to the plasma membrane to communicate with central signaling molecules including phosphatidylinositol-3 kinases (PI3K), mitogen-activated protein kinases (MAPK), focal adhesion kinase (FAK), protein kinase B/AKT, and protein kinase C. These effectors subsequently govern activation, growth, proliferation, and survival [16, 24].

## Features of HEM1 Deficiency

### Genetics

**Fig. 2:**
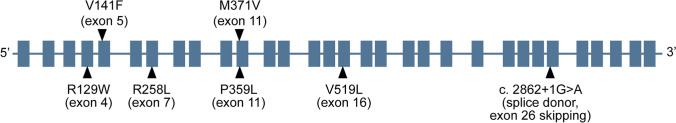
Exon map of *NCKAP1L* gene with locations of all reported pathogenic variants

HEM1 deficiency was first described in 2019 and has been reported in nine individuals from seven kindreds [8–10, 25]. In the Genome Aggregation Database (gnomAD), the observed to expected ratio of loss-of-function (LoF) variants in *NCKAP1L* was 0.20 (90% CI 0.13–0.33), suggesting there is selection against HEM1 LoF [26]. All reported pathogenic variants in the 31-exon *NCKAP1L* gene on chromosome 12 (NM_005337.5) segregated with disease and followed autosomal recessive inheritance patterns (Fig. 2). Most are biallelic missense variants resulting in destabilizing amino acid substitutions: p.R129W in exon 4, p.V141F in exon 5, p.R258L in exon 7, and p.P359L and p.M371V in exon 11 [8, 9]. Two siblings carried compound heterozygous deleterious mutations encoding the p.P359L variant as well as a p.V519L variant in exon 16 [8]. None of the variants is present in the Exome Variant Server and only two variants (c.1076C > T [p.P359L] and c.1111A > G [p.M371V]) are observed in gnomAD, though there are no healthy homozygotes [26]. HEM1 protein modeling suggested that the affected amino acids, despite their locations on distant exons, cluster in a hotspot that is likely crucial for conformational stability [16]. These HEM1 amino acid substitutions destabilize the entire complex, causing subsequent degradation of all WRC subunits. Loss of all WRC components was also observed in cells from a patient who harbored a homozygous splice site mutation (c.2862 + 1G > A) resulting in exon 26 skipping [10]. By contrast, the p.M371V substitution drastically reduced WRC activation by the upstream GTPase ARF1, thus impairing WRC function without destabilizing the complex [8].

### Clinical Features

HEM1-deficient patients typically present in the first year of life with dysregulated immune responses leading to a syndrome of immunodeficiency coupled with hyperinflammation, lymphoproliferation, and autoimmunity (Table 1). While the patient cohort reported by Salzer et al. displayed a predominantly autoimmune phenotype and those reported by Castro et al. had features consistent with hemophagocytic lymphohistiocytosis (HLH), the patients reported by Cook et al. had a mixed clinical picture, highlighting the overlapping features and commonalities. All patients suffer from recurrent infections such as otitis media, upper respiratory infections, pneumonia, and abscesses [8–10]. Respiratory infections commonly lead to chronic complications including bronchiectasis. Interstitial lung disease is also described without a clear etiology but may be related to viral infections or chronic inflammation [8, 27]. Other bacterial infections have included cellulitis, septic arthritis, and cholecystitis [8–10]. Reported viral infections have included disseminated herpes simplex virus, Epstein-Barr virus viremia, and gastroenteritis [8–10]. One patient has been described with lymphadenitis after Bacillus Calmette-Guerin vaccination, but this occurred after initiation of steroids [10]. Notably, siblings of patients from three of the seven kindreds died before 3 years of age from unknown causes or known infections, possibly because of HEM1 deficiency [8, 9].

**Table 1:** Clinical and laboratory characteristics of patients with HEM1 deficiency

Patient information	Pt. 1.1	Pt. 2.1	Pt. 2.2	Pt. 3.1	Pt. 4.1	Pt. 5.1	Pt. 5.2	Pt. 6.1	Pt. 7.1	Cohort
Age at symptom onset	9 months	6 months	11 Months	13 Months	12 months	4 months	12 months	1.5 months	5 years	
cDNA mutation (protein change)	c.1076C > T (P359L)	c.1076C > T/c.1555G > C (P359L)/(V519L)	c.1076C > T/c.1555G > C (P359L)/(V519L)	c.1111A > G (M371V)	c.773G > T (R258L)	c.385C > T (R129W)	c.385C > T (R129W)	c.421G > T (V141F)	c.2862 + 1 G > A (splice site skipping exon 26)	
Clinical features										
Recurrent infections	+	+	+	+	+	+	+	+	+	9/9
Lymphadenopathy	+	+	+	-	-	-	+	-	-	4/9
Hepatomegaly	+	+	+	+	+	-	-	-	+	6/9
Splenomegaly	+	+	+	+	+	-	-	+	+	7/9
Atopic disease (allergy, asthma, excema)	+	+	+	+	+	-	-	-	-	5/9
Laboratory features										
IgA	nl	Low	nl	nl	nl	nl		High	High	High 2/9; low 1/9
IgG	High	High	High	nl	nl	nl		High	High	High 5/9
IgM	nl	Low	low	nl	High	nl		High	High	High 3/9; low 2/9
IgE	High	nl	High		High			High	nl	High 4/9
T cells										
ab DNT	High	High	nl		nl	nl	nl			High 2/9
CD4 naïve	nl	Low	Low			Low	Low	Low	Low	Low 6/9
CD4 central memory	nl					nl	High	High	High	High 3/9
CD4 effector memory	nl					High	nl	High	High	High 3/9
CD4 TEMRA						High	High	nl	nl	High 2/9
CD8 naïve	nl	Low	Low			nl	Low	Low	Low	Low 5/9
CD8 central memory	nl	High	High			High	High	nl	High	High 3/9; low 2/9
CD8 effector memory	nl					nl	nl	nl	High	High 1/9
CD8 TEMRA	High					Low	Low	High	High	High 3/9; low 2/9
CD4/CD8 ratio	nl	Low	Low	nl				Low	Low	Low 4/9
B cells	High	High	High	nl	nl	High	High	High	nl	High 6/9
NK cells	Low	Low	Low	nl	nl	nl	nl	nl	nl	Low 3/9
Autoantibodies	-	-	+	-	-	+	-	+	-	3/9
Poor antibody response to vaccination or infection	-	+	+	+	+			+	+	6/9
HLH	-	-	-	-	+	-	-	+	+	3/9
Reference	Cook and Comrie et al. [8]					Salzer and Zoghi et al. [9]		Castro et al. [10]		

*DNT*, double negative T cells; *TEMPRA*, T effector memory RA+; *NK*, natural killer cells; *PC*, pneumococcal; *HLH*, hemophagocytic lymphohistiocytosis; *nl*, normal; *blank*, data unavailable

Autoimmunity and hyperinflammation are commonly observed in HEM1-deficient patients. Patients may have systemic lupus erythematosus–like disease with positive anti-nuclear antibodies (ANA) and double-stranded (ds) DNA autoantibodies, and associated immune complex glomerulonephritis [8–10]. Episodes of fever can occur in conjunction with lymphadenopathy or hepatosplenomegaly. Autoimmune thrombocytopenia in one patient occurred after measles, mumps, and rubella vaccination [8]. Atopic disease in the form of food allergy, allergic rhinitis, and asthma occurs in more than half of the patient cohort, highlighting exaggerated atopic inflammatory responses against environmental allergens and irritants [8–10]. Three patients with features such as fever, splenomegaly, hyperferritinemia, hypertriglyceridemia, hypofibrinogenemia, and increased soluble IL-2 receptor met diagnostic criteria for hemophagocytic lymphohistiocytosis (HLH) [8, 10, 28, 29] and demonstrated transaminitis or sinusoidal dilatation on liver biopsy [8, 10]. One patient later developed hepatic fibrosis (data not shown). Lymph node biopsy from a different patient showed evidence of histiocytosis and emperipolesis suggesting Rosai-Dorfman disease [8].

Other clinical features include mild microcytic anemia and anisopoikilocytosis [10]. Interestingly, several dysmorphic and developmental syndromic features may be observed, such as intracerebral ventricular dilation, tricuspid valve deficiency, bicuspid aortic valve, ventricular septal defects, pectus carinatum, and poor dentition [8]. Failure to thrive was noted in two patients [8, 9].

### Immunophenotype and Serologic Findings

Immunophenotypes in affected individuals have been variable (Table 1). Most patients (six out of nine) displayed increased total B cells [8–10]. Increased proportions of naïve B cells and B cell subsets associated with autoimmunity (innate-like memory B CD19^+^CD21^lo^CD38^lo^ and CD19^+^CD21^−^) were observed less consistently [9, 10]. Patients with severe autoimmune features display reduced proportions of naïve CD4^+^ and CD8^+^ T cell populations [8–10]. In contrast, subsets of CD4^+^ central memory (CM), effector memory (EM), and T effector memory RA^+^ (TEMRA) cells are frequently increased [9, 10]. Naïve CD8^+^ T cell populations were low, and no consistent pattern has been observed with CD8^+^ memory cells [8–10]. One investigation revealed increased exhaustion and senescence in patient CD4^+^ and CD8^+^ T cells, closely resembling the phenotype of DOCK8-deficient T cell [10, 30]. Two unrelated patients displayed persistently elevated proportions of αβ double-negative T (DNT) cells, an interesting comparison to the hallmark expansion of DNTs in autoimmune lymphoproliferative syndrome [8, 31, 32]. Natural killer (NK) cells were either normal or decreased across the cohort [8–10]. Subsequent investigations of immune cell population skewing were hampered by the use of immunosuppressive medications (see Therapeutic Perspectives). While immunophenotypes at disease presentation vary significantly, functional HEM1 is required for balanced lymphoid lineage hematopoiesis. This diversity of changes may be due to alterations of lymphocyte ontogeny and mature cell differentiation, as well as differences in genetics, epigenetic changes, and pathogen exposures.

Patients have variable immunoglobulin abnormalities. Elevated IgE is frequently observed, especially in those with asthma or atopic symptoms [8, 10]. More than half of the patients had elevated IgG levels without a clear relationship to autoimmune manifestations [8, 10]. One patient displayed selective IgA deficiency in the context of celiac disease while others displayed mildly elevated IgA levels [8, 10]. Impaired specific antibody production after vaccinations or EBV infection was observed in six patients [8, 10].

## Proposed Pathogenic Mechanisms

**Fig. 3:**
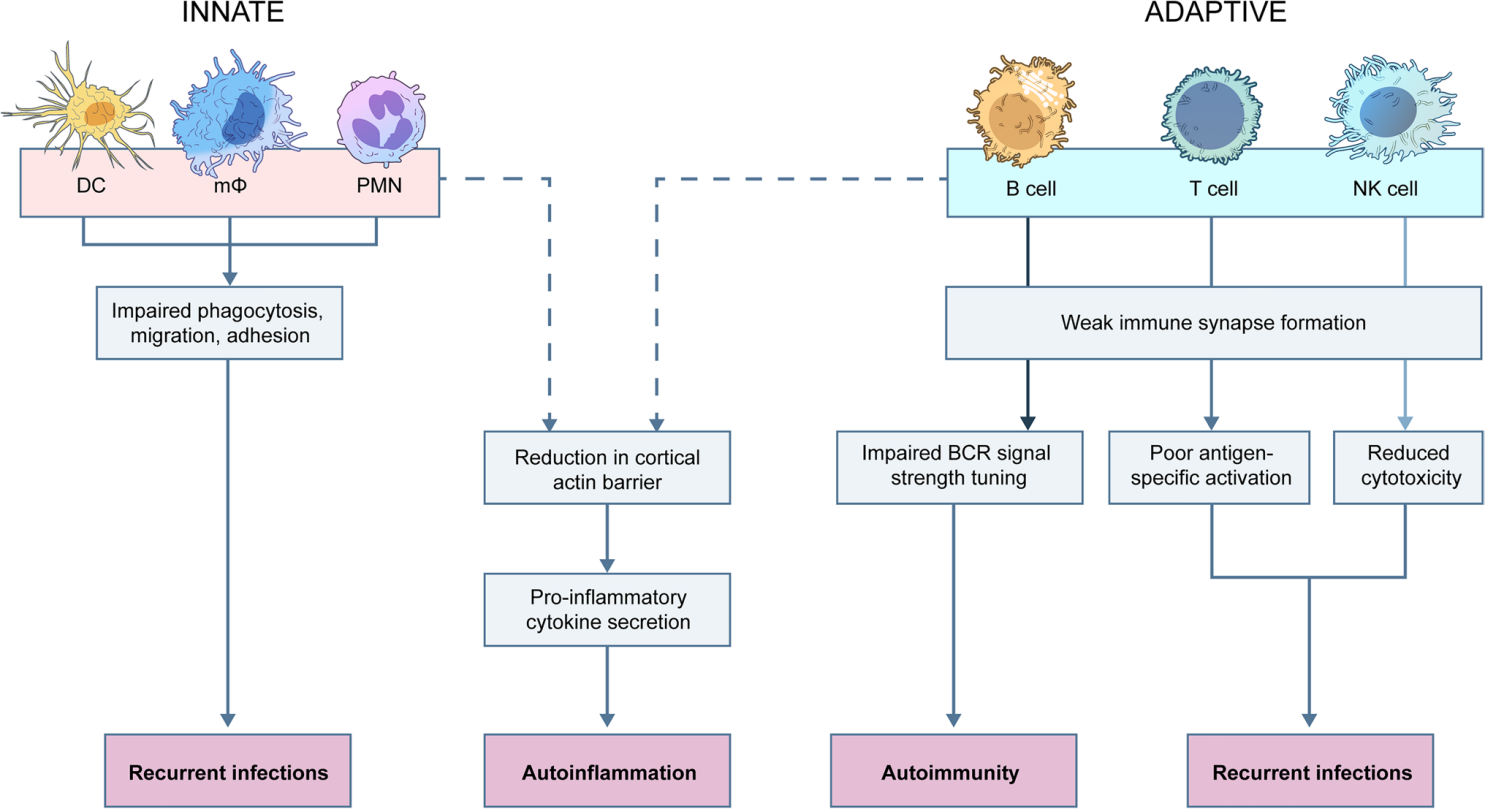
Schematic of cellular defects associated with HEM1 deficiency and their associated clinical manifestations

Various mechanisms have been proposed to explain the array of abnormalities and clinical features observed in HEM1-deficient patients (Fig. 3), but gaps in our knowledge remain. Like mouse models of HEM1 deficiency, defects in neutrophil, dendritic cell, and macrophage migration and phagocytosis impair innate immune defenses, predisposing patients to bacterial infections [32–35]. Interestingly, myeloid-specific knockout of Hem1 in mice increased susceptibility to infection and morbidity upon influenza A and *Streptococcus pneumoniae* challenge [36]. Poor pathogen clearance, accumulation of debris, and excess cytokine production by alveolar macrophages may predispose patients to recurrent infections and long-term lung injury.

Because T cell immune synapses are highly dependent on ARP2/3-mediated F-actin polymerization [12, 22, 23, 37], HEM1-deficient T cells fail to properly integrate antigen signals and fully activate. This manifests as reduced CD25 and CD69 upregulation and poor proliferative responses in patient cells after TCR engagement in vitro [8, 9]. Similar defects in B cell immune synapses likely underlie poor antibody responses after vaccination or infection [9, 38]. Ultimately, insufficient innate and adaptive responses culminate in recurrent infections in HEM1-deficient patients.

Autoimmune features may result from impaired B cell receptor (BCR) signal strength during germinal center reactions leading to autoantibody production. During B cell development and BCR editing, defective actin polymerization at immune synapses results in weaker signals, allowing for survival of autoreactive B cells with inherently stronger signals [9, 39, 40]. Numerous Hem1-deficient mouse models demonstrated systemic autoimmunity and inflammation parallel to that seen in patients, including splenomegaly, immune complex lupus glomerulonephritis, elevated proinflammatory markers (IL-6, IFNγ, and MRP8/14), and autoreactive plasmablasts with elevated serum dsDNA autoantibodies [9, 41].

Prolonged pathogen exposure resulting from impaired adaptive immune cell activation is known to contribute to chronic inflammation and HLH development, potentially explaining the HLH-like phenotypes observed in patients [28, 42]. Despite failure to properly initiate adaptive responses, in vitro studies of activated patient CD8^+^ T cells revealed hypersecretion of cytotoxic cytokines, including perforin and granzymes A and B [8]. Knockdown of HEM1 in pan-T cells and subsequent reactivation resulted in increased levels of soluble granzymes, interferon gamma (IFNγ), IL-4, and IL-6, corresponding to increased serum levels of IFNγ and IL-6 observed in Hem1^−/−^ mice [8, 9]. Multiple investigations found elevated surface CD107a levels after CD8 + T cell degranulation and lower cortical actin thickness, indicating that reduced integrity of the mechanical actin barrier facilitates vesicular hypersecretion [8, 10, 12, 33]. This draws an interesting comparison to the normal or elevated CD107a levels observed in the form of familial HLH in which granules lack functional perforin [28]. Diminished cortical actin barriers could also explain the observation that patient NK cells had higher basal degranulation rates [10]. An overall proinflammatory environment in secondary lymphoid organs, despite impaired specific lymphocyte activation, may explain the hepatomegaly, splenomegaly, and lymphadenopathy that was observed broadly across patient cohorts.

### WRC and mTOR

Regulation of the cytoskeleton by mechanistic target of rapamycin (mTOR)–containing complex 2 (mTORC2) is well documented. It was initially shown that mTORC2 modulates cell morphology via PKCα signaling [43]. mTORC2 is activated by increased plasma membrane tension as part of a negative feedback system to limit actin-based cellular protrusions, thus strengthening the notion that mTORC2 is involved in mechanosensory cytoskeletal signaling cascades influencing cell shape and motility [44]. The investigation of HEM1-deficient patient cells and mouse models offers a unique insight into the relationship between mTORC2 and actin polymerization. Phosphorylation of AKT at serine 473, a specific target of the mTORC2 kinase complex, in response to various stimuli (CD3/28 TCR stimulation, IgM BCR stimulation, fibronectin, and ICAM-1) was diminished in T and B cells from patients [8, 9]. Impaired AKT activation and downstream dysregulation of Foxo1 substrates in *Hem1*-/- mouse B cells likely contributes to defects in proliferation and survival. Interestingly, a model of T cell–specific *Wave2* knockout in mice produced the opposite effect; these mice displayed autoimmunity and cytokine hypersecretion similar to patients, but loss of WRC components resulted in enhanced mTORC1 and mTORC2 activity in mice [41]. These paradoxical findings suggest that distinct defects across multiple WRC-deficient hematopoietic lineages, rather than defects isolated to T cell lineages, interact to create a complex, systemic disease in humans. The exact mechanism of crosstalk between these two critical complexes is not fully understood; however, physical interaction between WRC and mTORC subunits was observed, suggesting direct modulation of kinase or actin polymerization activity [8, 41].

## Biological Findings and Diagnosis

Initial clinical consideration of HEM1 deficiency may be challenging due to the clinical heterogeneity at presentation, as well as the phenotypic overlap with other known actinopathies and lymphoproliferative disorders. Findings of recurrent infections, poor responsiveness to vaccination, secondary lymphoid expansion, and serology suggestive of autoimmunity in the first year of life coupled with a negative genetic workup for autoimmune lymphoproliferative syndrome (ALPS) should prompt consideration of HEM1 deficiency as an alternative diagnosis [45]. Likewise, HEM1 deficiency should be ruled out in children presenting with hemophagocytic lymphohistiocytosis (HLH)–like autoinflammatory features [29]. The increasing availability of whole exome and genome sequencing in the diagnostic process should lower the threshold to initiate genetic testing in children with immune dysregulation. Initial investigations that should be considered, perhaps in collaboration with an immunology research laboratory, include immunoblotting to assess for WRC destabilization, though this test would have poor sensitivity in detecting variants that inhibit WRC activation without affecting protein levels. Supplementary tests may include T and B cell activation studies and neutrophil migration assays.

## Therapeutic Perspectives

With the limited number of reported cases, proper therapy is still being defined. Corticosteroids and other immune suppressants such as sirolimus were clinically effective in controlling lymphoproliferation, autoimmunity, and auto-inflammatory symptoms. It is worth noting that using sirolimus is counterintuitive given in vitro data suggesting that HEM1 deficiency reduces mTORC activity [8, 41]. While this remains an area for investigation (see below), the clinical benefit of sirolimus in some patients is possibly attributable to reduced immune cell activation and proliferation in pro-inflammatory settings. Clinical responses to steroids were variable; one patient was successfully weaned off, but most required chronic steroids, azathioprine, or mycophenolate mofetil for long-term immune suppression. Rituximab was initiated in one patient during an acute exacerbation of nephrotic syndrome, leading to partial resolution of renal disease. Immunoglobulin replacement and prophylactic antimicrobials should be considered to prevent recurrent infections. Given the young age at presentation, severity of infections, systemic inflammatory complications (i.e., interstitial lung disease, glomerulonephritis, hepatic fibrosis), and dependence on immunosuppressants, hematopoietic stem cell transplant (HSCT) is a consideration. HSCT is a known treatment for other hematopoietic actinopathies such as WAS and DOCK8 deficiency and would likely rescue HEM1 in all relevant hematopoietic cell lineages. However, there are no data currently available for outcomes of HSCT in HEM1-deficient patients, including in some follow-up discussions regarding reported patients.

## Areas for Investigation

Beyond blood, the HEM2-containing WRC has been broadly implicated in non-immunological diseases and is especially associated with tumor progression and metastasis in melanoma, non-small cell lung cancer, and breast cancer [46–48]. Additionally, neural tissues are highly dependent on cytoskeletal dynamics. Disruption of CYFIP2 and its interaction with Fragile X Mental Retardation Protein (FMRP) is known to cause a neurodevelopmental disorder characterized by intellectual disability and seizures [49]. Recently, WAVE2 was found to be directly modulated by leucine rich repeat kinase 2 (LRRK2) in microglia, establishing a potentially pathogenic role in development of Parkinson disease [50]. However, since HEM1 deficiency is the first reported WRC-related immunodeficiency, numerous mechanistic and clinical questions remain to be answered.

Immunoprecipitation and in vitro kinase studies showed that WAVE2 suppressed mTORC1 and mTORC2 activity [41]. However, data from patient cells reliably demonstrated reduced mTOR activity in the absence of WRC [8, 9]. Parsing out the intricate feedback loops between mTORC1 and mTORC2, and how the physical interaction of the WRC with mTORC impacts signaling cascades, will be necessary for understanding cellular defects in HEM1-deficient patients and the role of mTOR inhibitors in therapy. Furthermore, despite the instability of the WRC without each of its five subunits, some data suggest that HEM1 may exist in pools outside of the WRC. Determining the independent role of HEM1 could reveal novel pathways and additional therapeutic targets. A recent report demonstrated expression of an alternative *NCKAP1L* splice form in biliary epithelial cells promotes intrahepatic biliary tree morphogenesis [51]. These interesting findings should be validated, as extra-hematopoietic expression of *NCKAP1L* would have significant implications for disease pathogenesis and treatment.

As additional data become available from newly diagnosed patients, the full clinical picture of HEM1 deficiency will become clearer. Given the clinical heterogeneity at present, development of a clinical scoring system may assist in defining disease subtypes and determining genotype–phenotype correlations. Translational studies investigating immunomodulatory agents that limit inflammation and irreversible organ damage can drastically improve patient outcomes. Understanding the efficacy of HSCT will guide management, though the significant risks of rejection and graft-versus-host disease after HSCT make autologous gene-modified HSCT an appealing option [52, 53]. Ultimately, the best outcomes for patients with actin-related IEIs like HEM1 immunodysregulatory disorder will require early diagnosis and a precision medicine approach to management.

## Data Availability

Not applicable as a review paper, but primary sources utilized can be made available upon request.
